# Iron deficiency without anemia – a clinical challenge

**DOI:** 10.1002/ccr3.1529

**Published:** 2018-04-17

**Authors:** Esa T. Soppi

**Affiliations:** ^1^ Outpatient clinic Eira Hospital Laivurinkatu 29 Helsinki 00150 Finland

**Keywords:** Anemia, ferritin, hemoglobin, iron deficiency

## Abstract

One should always consider iron deficiency (without anemia) as the cause of persisting, unexplained unspecific, often severe, symptoms, regardless of the primary underlying disease. The symptoms of iron deficiency may arise from the metabolic systems where many proteins are iron containing. Long‐standing iron deficiency may be challenging to treat.

## Introduction

Iron deficiency may be severe despite a normal hemoglobin and full blood count. Symptoms which may be prolonged and debilitating, should raise a clinical suspicion on iron deficiency even if full blood count is normal. A lifelong history of blood loss, such as abundant menstruation, pregnancies, blood donations, accidents/surgery as well as history of celiac disease, atrophic gastritis and drugs limiting gastric acid secretion, should be taken. Ferritin (<30 *μ*g/L) is most sensitive and specific indicator of iron deficiency, although its pitfalls need to be taken into consideration. However, the ferritin concentration may be near to normal, while iron staining of a bone marrow aspiration sample is devoid of iron. Furthermore, in determining the iron status, it is essential not to rely only on results of a single test but to consider the whole picture. Iron therapy should be monitored with repeated ferritin determinations with a target ferritin concentration of >100 *μ*g/L and carried out until symptoms have resolved. When iron treatment is discontinued, the serum ferritin should be determined to ensure that the level remains stable. Iron should be reinstituted if the ferritin concentration drops and symptoms reappear.

### Clinical challenge

Iron deficiency without anemia is a diagnostic challenge, as it may go unrecognized for a longer period and furthermore, there are no well‐defined diagnostic criteria. The suspicion should arise, if a patient with normal full blood count presents symptoms of iron deficiency anemia 1, 2, 3 primarily together with low ferritin concentration and especially when the medical history supports iron deficiency.

Iron deficiency anemia (hemoglobin ≤ 130 g/L in males and ≤120 g/L in females) is a late manifestation of iron deficiency, both of which are common medical conditions in everyday clinical practice 2, 3, 4. Some 10–20% of menstruating women have iron deficiency, and 3–5% of them are frankly anemic 4. Iron deficiency (anemia) may often be asymptomatic and go undiagnosed for a long period of time. Blood is frequently drawn for a full blood count and if microcytic/hypochromic anemia is present, iron deficiency may be suspected. The serum ferritin concentration (cut off <30 *μ*g/L) is the most sensitive and specific test used for identification of iron deficiency 2, 3. However, in clinical laboratories, the lower limit of the reference range is often set at 10–20 *μ*g/L 5. This may lead confusion as the role of ferritin in the detection of iron deficiency is not unequivocal. Then the ferritin can be supplemented with determination of transferrin saturation, soluble transferrin receptor (sTfr) and the ratio between sTfr and logarithm of ferritin as well as hepcidin 2, 3.

### When is the ferritin concentration abnormal?

The question of which ferritin concentration signifies clinically symptomatic iron deficiency has been raised 5, 6. Patients with true iron deficiency anemia on the basis of negative bone marrow iron staining may have a serum ferritin concentration close to 50 *μ*g/L 7. Patients with the restless leg syndrome should be considered iron deficient when their ferritin concentration is <75 *μ*g/L 8. Furthermore, patients with negative bone marrow iron stores have been shown to present with serum ferritin levels of close to 100 *μ*g/L 9. Iron deficiency in patients with heart failure impairs the quality of life, irrespective of the presence of anemia. There, iron deficiency was defined as a ferritin level <100 *μ*g/L or, at 100–299 *μ*g/L with transferrin saturation <20%, 10. Notably patients with inflammation or clinically significantly impaired liver or renal function were excluded from the trial 10.

### Symptoms and differential diagnosis

Weakness, fatigue, difficulty in concentrating, and poor work productivity are nonspecific symptoms ascribed to low delivery of oxygen to body tissues and decreased activity of iron‐containing enzymes 2, 3, 11, 12, 13. The extent to which these non‐hematologic effects of iron deficiency are manifested before anemia develops may be unclear 2, 3, 4.

During my 30‐year carrier as an internist with a special interest in thyroid diseases and hematology, I have met hundreds of patients, mainly menstruating females, who seek advice because of prolonged (1–25 years) fatigue, brain fog, muscle and joint pains, weight gain, headache, dyspnoea, palpitations, sometimes associated with sleep disturbances, arrhythmia, lump in the throat or difficulty in swallowing, and restless legs. The patients have often received a spectrum of diagnoses, such as subclinical hypothyroidism (treated with levothyroxine alone or with T3 containing preparations), chronic fatigue syndrome, fibromyalgia, chronic Lyme disease, burnout, and overtraining. The blood count has usually been normal. At referral, their serum ferritin concentrations have ranged from 1 to about 150 *μ*g/L. If there has been no obvious reason for iron deficiency, such as celiac disease, multiple blood donations, multiple pregnancies, or long periods of abundant menstruation, the differential diagnostics at serum ferritin >50 *μ*g/L has included liver and kidneys diseases, occult blood in stools, IgA deficiency, calcium disorders, D‐vitamin or vitamin B12 deficiency and, if the history revealed prolonged use of pyridoxine (>20 mg/daily, vitamin B6 toxicity 14.

### Cases highlighting the problem

Over the past 10 years I have treated patients without anemia but symptoms of iron deficiency and borderline serum ferritin concentrations with oral (hundreds) and/or intravenous iron (over 200) with excellent results. Here are two examples.

*Patient 1*. A previously a healthy 55‐year‐old female (body mass index 24.8 kg/m^2^) was hospitalized 2 months earlier because of severe subacute thyroiditis (fever, severe neck pain, sedimentation rate 105 mm/h, C‐reactive protein 145 mg/L, free T4 35 pmol/L) and she was treated with prednisolone (starting 60 mg daily). At the first visit, 3 months after start of treatment, the patient was still taking prednisolone 10 mg daily. Her TSH was 21 mU/L and free T4 8.5 pmol/L. Levothyroxine 100 *μ*g/day was initiated but after few days and she reported severe palpitations and the levothyroxine dose was halved and later discontinued. At the same time, she reported muscle and joints pains already few years back and restless legs (occasional use of pramipexole 0.0088–0.018 mg/day) for 10 years. Her menopause had occurred 6 years earlier, but her menstruation had always been scarce and she had never donated blood or been pregnant. The hemoglobin concentration was 140 g/L but the ferritin concentration was 27 *μ*g/L; liver and kidney diseases as well as celiac disease was excluded and repeated tests for occult blood in stools were negative.

**Figure 1:**
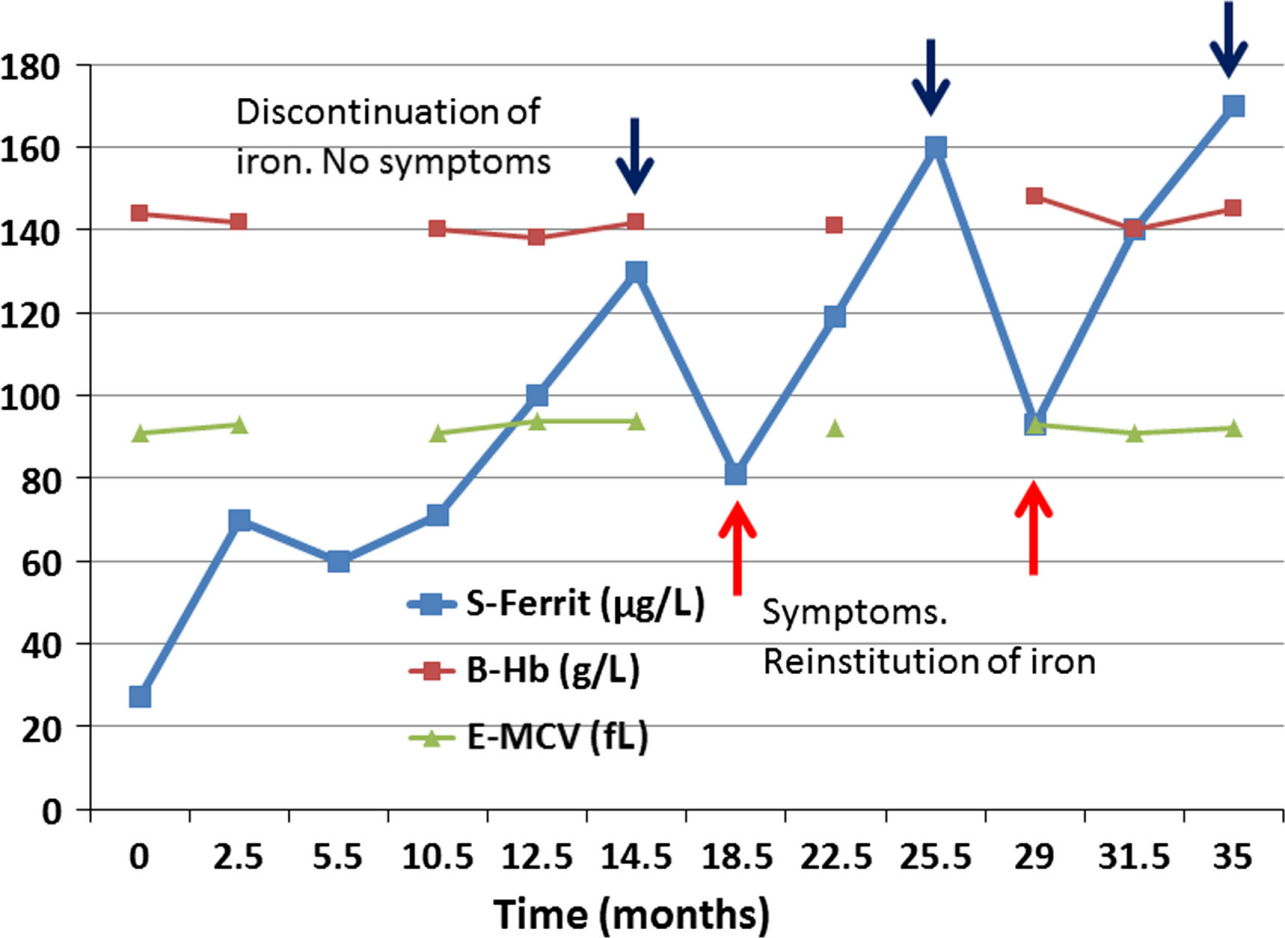
Hemoglobin, mean corpuscular volume (MCV) and ferritin of a patient, who received 1‐year oral iron and whose symptoms relapsed twice after discontinuation of oral iron at the same time when the ferritin concentration decreased to the <100 *μ*g/L.

The patient was considered iron deficient for reasons unknown. Ferrous sulfate 150 mg daily was prescribed. After 12 months the serum ferritin concentration was 100 *μ*g/L without any change in hemoglobin concentration, but her years‐long symptoms had gradually disappeared (Fig. 1). The iron supplementation was continued for another 4 months and then discontinued. Gradually the patient reported reappearance of restless legs and fatigue. Then full blood count and ferritin were determined and the iron supplementation was reinitiated. The same took place when the iron was again withdrawn without any change in the full blood count.

**Figure 2:**
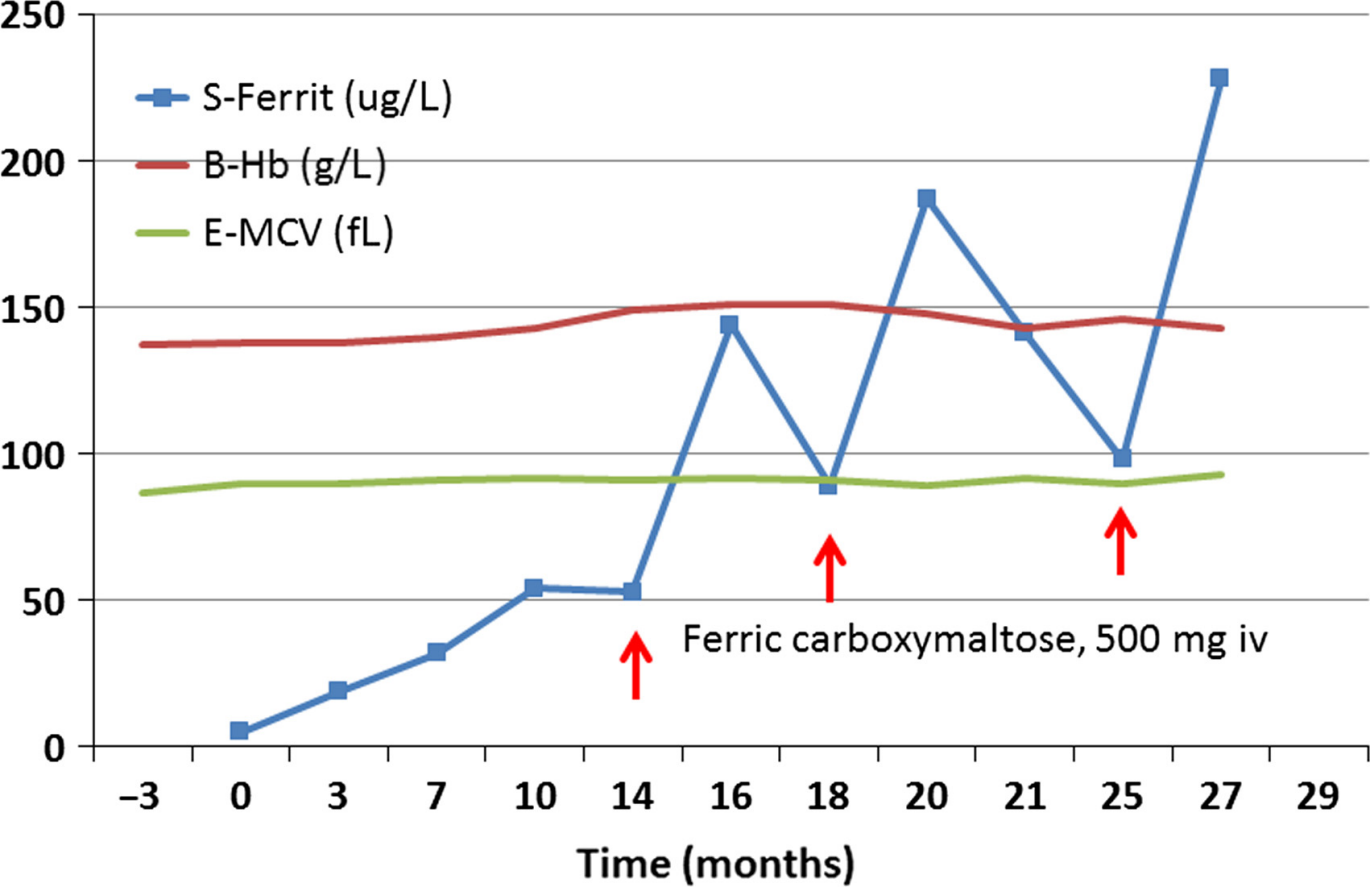
Hemoglobin, mean corpuscular volume (MCV) and ferritin of a patient who received oral iron for 1 year and thereafter intravenous iron three times after the first two infusions the patients' symptoms relapsed when her ferritin concentration decreased to the <100 *μ*g/L.

*Patient 2* was a healthy 40‐year‐old female (body mass index 31.6 kg/m^2^) but had always had abundant menstruation with duration of 5–7 days. She had had two normal pregnancies and deliveries 10 and 7 years earlier with a history of anemia during pregnancies. One year before referral levothyroxine treatment (100 *μ*g/daily) for hypothyroidism had been instituted (positive family history, TPO antibodies >1000 IU/mL, TSH 9,4 mU/L and free T4 9.9 pmol/L). The patient was referred because of intractable fatigue and sleep disturbances (waking up several times during the night for 6 years). Sleep apnea was excluded by polysomnography. The thyroid hormone had induced chemical euthyroidism and had relieved her fatigue slightly. Her history raised a suspicion of iron deficiency despite a normal complete blood count (hemoglobin concentration 137 g/L) 3 months before referral. The ferritin concentration was only 5.4 *μ*g/L (Fig. 2). Oral iron was prescribed with increasing doses and from month 7–14 the patient took ferrous sulfate 100 mg three times daily with apparently good compliance. During that time both her hemoglobin increased by 10 g/L as a sign of iron deficiency, the tiredness and sleep disturbances were somewhat relieved, but the patient was not still feeling well. Iron (ferric carboxymaltose, 500 mg) was infused i.v. on months 14, 18, and 25 and every time all the symptoms disappeared within 4 weeks after the infusion and reappeared, when the ferritin level dropped to about 100 *μ*g/L after the first two infusions. The patient has no knowledge of the laboratory results at the time, when she reported the reappearance of the symptoms.

## Discussion

Iron deficiency is a common clinical challenge 2, 3, 4, but the diagnosis is difficult when blood count is normal. It has been unclear to what extent the non‐hematologic effects of iron deficiency are manifested before anemia, if it develops 2, 3, 11. Even if ferritin is the most sensitive method to detect iron deficiency 2, 3 it may be difficult to determine which ferritin concentration is indicating iron deficiency 5, 6. Therefore one has to rely on non‐hematologic symptoms and interpret the ferritin concentration in conjunction with symptoms taking into consideration the differential diagnostic possibilities which may give similar symptoms. Iron deficiency is usually a result of increased host requirements such as high‐performance sport, limited supply such as vegetarian diet, and increased blood loss. Often this can be ascertained by a lifetime history of iron loss due to heavy menstrual bleeding, blood donations, multiple pregnancies, accidents/surgery among others 15. Furthermore, the diagnosis is supported by the ferritin determination and exclusion of other causes of symptoms 16, 17. Hypothyroidism based on symptoms is indistinguishable from iron deficiency 18, 19. Fatigue and neurocognitive symptoms often raise a suspicion of depression. Furthermore, headache and muscle and joint pain associated with iron deficiency are repeatedly considered migraine and fibromyalgia syndrome, respectively 3, 19. The multitude of symptoms is commonly associated low ferritin concentration without anemia 1, 17, 20, 21, 22. In addition, iron supplementation has been shown to improve fatigue and physical performance with low ferritin concentration 23, 24, 25. The symptoms of iron deficiency may arise from the metabolic system where many of the proteins are iron containing 2, 3, 11, 12, 13. Even if the amount of functional protein‐bound iron is minimal its role is apparently important. The conclusion is that the supply of iron to these systems may also explain the fluctuation of symptoms as described in the cases. Furthermore, there may a question of iron deficiency in combination with functional iron deficiency, where there is an insufficient mobilization of erythroid iron in the presence of increased requests 2, 3, 5.

Iron should be administrated much longer, on average 6–12 months, than only a few months, which is common practice in general care 3. This may partly due to poor compliance mainly due to adverse effects of oral iron and this should always be addressed during following‐up contact with the patients during oral iron therapy. Intolerance to oral iron is probably the main cause for intravenous iron therapy. Even if the severe adverse effects with the current intravenous iron preparation are rare 2, 3 they still exist and may lead to the discontinuation of the infusion, and strategies to treat them should be planned beforehand. Furthermore, it needs to be taken into consideration that in relation to intravenous iron there may be a placebo effect of improved subjective well‐being. In case 2, this is most probably not the cause of fluctuating symptoms, as the patients did not know the behavior of ferritin concentration when she reported the reappearance of her symptoms.

## Conclusion

The presented cases with symptoms of iron deficiency anemia as examples are demonstrating that a normal full blood count is common in association with low ferritin levels that would indicate iron deficiency. It is much more important to listen to the patient's description of his/her symptoms than to use the full blood count to rule out iron deficiency. If symptoms are in accordance with iron deficiency, the patient should be considered iron deficient at least up to a serum ferritin concentration of 100 *μ*g/L, or even much higher, if the patient has an inflammatory condition, kidney disease or fatty liver 2, 3. Iron deficiency irrespective of manifestation should always be treated (17).

The ferritin level should be controlled regularly during and after the iron administration with a sustained target ferritin of more than 100 *μ*g/L. A marked improvement or total disappearance of symptoms should decide the duration of iron treatment. If the patient has apparently had iron deficiency for more than 5–10 years, the ferritin concentration may repeatedly drop with the reappearance of symptoms when oral (Fig. 1) or intravenous (Fig. 2) iron therapy is discontinued.

When the patient is symptomless he/she should be followed for an extended period of time to ascertain that the ferritin concentration remains stabilized, especially if the patient is a female with abundant menstruations or planning pregnancy. No blood donations should be allowed. I consider the diagnosis and management of iron deficiency without anemia as one of the greatest challenges during my 35‐year career as an internist. Furthermore, I am convinced that there is still a lot to be discovered about iron metabolism.

## Conflict of Interest

The author declares no conflict of interest.

## Authorship

ETS: solely responsible for examining and treating the patients and writing this study.
